# Empowered, Yet Vulnerable: Motives for Sport Participation, Health Correlates, and Experience of Sexual Harassment in Female Combat-Sport Athletes

**DOI:** 10.3390/sports10050068

**Published:** 2022-04-29

**Authors:** Therese Fostervold Mathisen, Radhika Singh Kumar, Kethe M. E. Svantorp-Tveiten, Jorunn Sundgot-Borgen

**Affiliations:** 1 Faculty of Health, Welfare and Organization, Østfold University College, 1671 Fredrikstad, Norway; 2 Department of Nutrition, Institute of Basic Medical Sciences, University of Oslo, 0372 Oslo, Norway; radhika.kumar8@gmail.com; 3 Department of Sports Medicine, Norwegian School of Sport Sciences, 0863 Oslo, Norway; kmengen@nih.no (K.M.E.S.-T.); jorunn.sundgot-borgen@nih.no (J.S.-B.)

**Keywords:** weight regulation, eating disorders, body composition, bone mineral density, body appreciation, energy availability, sexual harassment

## Abstract

Background: To explore motives for combat sport participation, weight regulation practices, symptoms of low energy availability (LEA), disordered eating (DE) or eating disorders (ED), and any experiences with sexual harassment (SH) among female combat-sport athletes. Methods: In total, 29 athletes were recruited by social media and in clubs. Participants responded to a questionnaire on health behavior and mental health and completed diet registration and a DXA-scan. Results: Most athletes started combat sports to feel empowered and experienced an inclusive milieu, but the frequency of health issues was high. A total of 21–67% had symptoms of ED, suffered from injuries, had low site-specific BMD, and/or symptoms of LEA. Athletes had insufficient intake of energy and nutrients, and <50% received any dietary information or guidance from their clubs. Most athletes complied with favorable weight-loss strategies; still, >20% used unfavorable methods and rapid weight-loss periods. A total of 70% of the athletes had experienced SH, of which 41% experienced SH within the combat-sport context. Conclusion: Combat sport offers an inclusive milieu, which may increase women’s health and confidence; still, our results indicates a need for actions to safeguard female combat-sport athletes’ mental and physical health, implying a cultural change within the community of combat sport and a need for increased health and nutrition literacy.

## 1. Introduction

Combat sports have historically been dominated by males and considered masculine according to traditional social construction [1,2]. While some women have practiced these sports for years, it was not until 2012 that women were allowed to compete in boxing in the Olympics, and a female first signed a professional contract in MMA within the UFC (Ultimate Fighting Championship, which is the premier mixed martial arts, MMA) [1]. This may be viewed as hallmarks for the acceptance for and increased interests in females practicing and competing within these sports [1], which concurrently challenges the socially constructed gender norms [1,2,3]. Intriguingly, the motives among female combat-sport athletes to engage in these sports has not been well explored. Combat sport (also here including most martial arts) revolves around physical self-defense techniques or also challenging an opponent for a fight, which contrasts the cultural expectations of females to not engage physically but on the contrary to uphold society’s perception of them as vulnerable and physically weak individuals [3]. The increased popularity of combat sport among females and the inclusion of females into sports with direct physical contact (e.g., MMA and professional boxing) in an arena and culture normally characterized as male-dominated have introduced potential dilemmas. Mixed-sex training may be difficult because athletes are concerned about inappropriate touches [4]. The nature of combat sport, i.e., the concentrated training of techniques, the one-to-one training, the many opportunities for intimate contact, and the minimal or tight clothing in some combat sports, may also enable coaches or co-athletes to exploit their position to gain physical or sexual contact, which may be perceived as unwanted and as harassment by the offended. Sexual harassment (SH) is by the literature and by law defined as “any undesirable sexual attention which is experienced as offensive, frightening, hostile, degrading, humiliating or troublesome” [5,6]. Motives for SH can be understood as an experience of threat to the traditional and cultural sex-related hierarchy [1], knowing that SH often occurs when the perpetrator has a higher hierarchical position [7]. As such, in a male-dominated milieu, the “intruding” females may experience inappropriate behavior by males as the male strives to maintain his hierarchical position. The four-factor theory, which rests on four explanatory elements, may support such an assumption. First, the perpetrator is motivated for SH (e.g., driven by power or sexual attraction); secondly, experiences of internal inhibitions are low (overcomes any moral restrains); third, there is a lack of external inhibitions (such as no clear communications on measures towards SH and/or emphasizing gender equality); and finally, the victim reflects low assertiveness or organizational position [8]. While some athletes and sport clubs within combat sport seem to picturize a culture in which casual sexual intercourse is common [4], the frequency of SH is not known. A previous publication reporting incidence of media reports on SH in combat sport raises concern due to the significant number of convicted sex offenders acting as coaches and resuming martial arts coaching following initial law enforcement intervention [9].

Combat-sport competitions are arranged and categorized according to body weight in order to justify the conditions of competition, and as such, competing in combat sport often means frequent weight regulation. A common practice is to reduce body weight prior to weigh-in to qualify for a lower weight-class and then regain body weight prior to competition, creating an obvious physical advantage [10,11]. Considerable and rapid weight fluctuations in combat-sport athletes have frequently been reported in the literature [11,12,13], and many of the techniques are harmful and life-threatening [12,14]. Research on the weight-regulation practices within these sports are concerned about the potential negative effects on physical performance and health due to the long-term effects from repeated weight fluctuations or prolonged periods of low energy availability (LEA), which also increases the risk of eating disorders (EDs) [14,15,16]. While some argue that female combat sport athletes are more prone to these negative health impairments compared to males [16], most studies are limited by few included females [15]. Importantly, it has been shown that persons with high level of body appreciation (i.e., “accepting, holding favorable opinions toward, and respecting the body, while also rejecting media-promoted appearance ideals”) are less prone to body figure idealization and the negative mental health effects from LEA [17,18,19]. A few studies have suggested poor body image and symptoms of or increased risk for EDs among female combat-sport athletes [10,15,20]; however, the frequency of disordered eating (DE) or EDs per se is not well-explored, and neither is body appreciation.

The objectives for this study was to explore motives among females to participate in combat sport; to evaluate how they practice weight regulation and if there are any symptoms of LEA, DE, or ED; and to study their experiences with sexual harassment within these gendered sports.

## 2. Materials and Methods

### 2.1. Design

This is a cross-sectional study aiming to explore motives for combat sport, sporting and health behavior (i.e., routines for weight regulation, dietary intake, levels of regular physical activity), and body weight and composition in competitive female combat-sport athletes. We also included questionnaires on mental health, specifically evaluating body appreciation, symptoms of EDs and of LEA, and experiences with SH.

### 2.2. Participants

**Figure 1:**
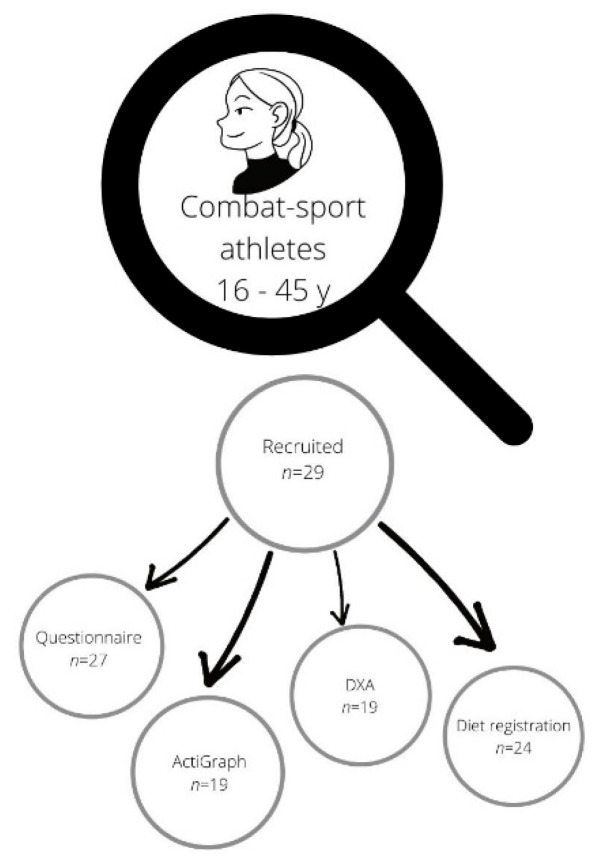
Total number of recruited and number of respondents to the four measurements performed. Discrepancies in numbers for each measurement are mainly due to COVID-19 pandemic society-restriction challenges.

We recruited active female combat-sport athletes face-to-face or by posters and folders handed out at the training facilities in combat-sport clubs during autumn 2020 (Figure 1). Those interested were given detailed information about the study and consented for participation by electronic signature. Important to notice is the concurrent COVID-19 pandemic during the recruitment period, which possibly influenced the numbers active at clubs, the numbers recruited, and the numbers of athletes not participating in competitions at that time.

### 2.3. Outcomes

An electronic questionnaire was designed with specifically formulated questions on background information regarding physical training history, motives for combat-sport involvement, sport-specific weight-regulation strategies, experiences of SH, and validated questionnaires on body appreciation, low energy availability, and symptoms of ED. We also included questions on how the COVID-19 pandemic affected their training and diet, as data sampling occurred concurrently to the national societal restrictions and measures. All were asked to wear an activity-registration device to register levels of habitual physical activity for 7 consecutive days and to register their diet for 4 days (3 weekdays and 1 weekend day). Additionally, all were invited to the laboratory of the Norwegian School of Sport Sciences to evaluate body composition and bone mineral density (BMD) by DXA.

Motives for combat sport involvement.

Participants were asked by open question to explain their motivation for joining the sport. Answers were coded into 5 different categories: empowerment (wanted to get stronger, wanted to learn self-defense, wanted to cope in a sport dominated by men, wanted to become self-confident and strong), friends (friends asked them to try/join in), group cohesion (good milieu, feel accepted and included), to become fit, or just by coincidence (noticed a recruitment campaign, wanted to try something different, was the only sport in the local area).

The Body Appreciation Scale, version 2 (BAS-2).

BAS-2 measures body appreciation, specifically how respondents value their body and their level of orienting cognitive processing to protect and promote a positive view of the body [19]. It consists of 10 items where participants respond to a Likert scale ranging from 1 (never) to 5 (always) and where a higher average score indicates a higher level of body appreciation [19]. Internal consistency (α) value of the BAS-2 was 0.92.

The Low Energy Availability for females Questionnaire (LEAF-q).

The LEAF-q screens for low energy availability (LEA) in female athletes and identifies the occurrence of injuries, gastrointestinal dysfunction (GD), and menstrual irregularities (MI) [20]. It has optimal sensitivity and specificity to identify LEA, reproductive function, and bone health in female endurance athletes and dancers [20]. Suggested cut-offs for GD, MI, and total LEAF-Q scores are ≥ 2, ≥ 4, and ≥ 8, respectively, with higher scoring indicating higher severity. Only those with no use of hormonal contraceptives (*n* = 10, 35%) were evaluated on MI. We defined symptoms of amenorrhea if menstrual bleedings had been absent for 3 months or more.

The LEAF-q has shown less specificity with higher total LEAF-q scores (i.e., ≥8) in a mixed sport cohort [21]. Therefore, it has been suggested to use the total-score cut-off to eliminate those with very low scores (i.e., <8) and to evaluate those above the cut-off more specifically [21]). The α value for the LEAF total score, GD, and MI ranged from 0.23 to 0.30.

The Eating Disorder Examination Questionnaire (EDE-q).

The EDE-q comprises 22 items scored 0–6 to measure the presence and 6 items scored openly to measure the frequency of core ED pathology [22]. It results in a global score (average score; 0–6) and four subscale scores (body weight concern, body shape concern, eating concern, and eating restriction) in which a higher score means higher level of clinical severity. The α value of the EDE-q global score was 0.94.

Body composition by Dual Energy X-ray absorptiometry (DXA).

Body composition was measured for 19 of 29 athletes, namely those who met for physical health evaluation at our lab. Participants were weighed in their underwear, and their height was measured with a fixed stadiometer (Seca scale, Mod: 8777021094, S/N: 5877248124885, Seca Deutschland, Hamburg, Germany). A DXA (Lunar iDXA, enCORE Software, version 14.10.022; GE Healthcare, Madison, WI, USA) performing a three-site scan (lumbar area (L2–L4); proximal femur (femoral neck, trochanter, and shaft); whole body) was used to measure body composition (fat mass (kg), percent body fat (%BF), lean body mass (kg), and BMD for spine and femur). All measures were done by one of two trained technicians, and all data were analyzed by one technician according to the guidelines [23]. Z-scores are normative values according to gender and age [24]; however, as athletes (and specifically those engaged in high-impact sports) are identified with higher bone mineral accrual [25] resulting in 5–15% higher BMD compared to non-athletes [26,27], we defined low bone mass by Z-score < 0 [28].

Levels of Physical activity by ActiGraph.

Levels of physical activity were objectively measured for seven consecutive days using the ActiGraph accelerometer (ActiGraph GT3x1; ActiGraph, LCC., Pensacola, FL, USA) placed on their right hip. The accelerometer was only removed for water activities and nighttime sleep. All accelerometers extract data from the vertical axis in 60 s epochs with 30 Hz sampling rate, with results presented as counts per minute (CPM). Non-wear time was determined as continuous zero count epochs lasting at least 60 min (allowing for two exceptions). Wear days were deemed valid if worn for at least 600 min/day with a minimum of two valid days.

Dietary intake.

All participants received oral and written information on how to register their diet for four days (three weekdays and one day during the weekend). They were instructed to register all food, fluids, and supplements consumed per meal, with details on type of food, volume of food, and time for meal intake. Volume was registered as household portions (e.g., a cup, a spoon, a slice, one item, etc.). Additionally, a picture of each meal was uploaded through a specially designed app “Nettskjema Bilde” (by Monica Hauger Carlsen, Ph.D., Institute of Nutrition, University of Oslo). A registered dietitian analyzed all diet registrations by use of a national dietary coding and analytical system (coding system KBS version 7.4, and the analytical system AE-18). We specifically extracted information on energy intake, intake of proteins, carbohydrate, fats, and fiber and intake of calcium, iron, and vitamin D and compared them to national recommendations and recommendations for athletes [29,30]

Sexual harassment.

First, SH was defined to respondents as “any undesirable sexual attention which is experienced as offensive, frightening, hostile, degrading, humiliating or troublesome”, similarly as defined by the Norwegian legal regulation (i.e., the Norwegian Equality and Anti-Discrimination Act) [6]. Secondly, participants were presented to dichotomous response alternatives (yes/no) to four separate questions on “experiences of unwanted sexual innuendos” (having any experience, experienced within the sport context, experienced outside the sport context, experienced within the sport context the last 12 months, respectively). Additionally, any experience of such SH within the sport context, during the last 12 months, or previously in life was followed up with questions on how often (once, a few times, often/regularly) and by whom (friends, teammate, coach, medical team members, family members, other).

Statistics.

All analyses were conducted in SPSS version 27 (IBM, Armonk, NY, USA). Data were visually inspected for normality and presented as mean (StD) if normal distributed or as median (range) if being non-parametric. We performed multiple regression analyses to examine possible explanatory factors for BMD Z-scores (total, hip, and spine) and EDE-q global score. We included two independent variables for BMD Z-scores (total exercise volume and past or present ED diagnosis) and three independent variables for EDEQ global score (BAS-2, present ED diagnosis, and total exercise volume). BMI was included as an adjustment variable. The variance inflation factor (≤5.0) was investigated, and no violations of cut point existed (30). Results from the linear regression analyses are presented as standardized coefficients (β), standard error (SE), and adjusted r^2^.

## 3. Results

In total, 29 female combat-sport athletes participated in this cross-sectional study, of which most were kick-boxers (*n* = 13, 45%). The others were (in falling order): five (17%) taekwondo athletes, five (17%) Thai boxers, four (14%) boxers, three (10%) karate athletes, two (7%) Brazilian Jiu Jitsu athletes, and two (7%) reported other sports (mixed martial arts and Kung Fu). Of these, six (21%) reported to participate in more than one sport. The majority competed at novice/national level, but two athletes were competing at the national level, and two athletes reported to be professional. In total, 20 (69%) had placed at the top three in national competition (NC), six (21%) had competed at a more recreational level, and three (10%) did not respond to this question. The mean (StD) years with specialized combat sport training were 6.3 (3.2) years.

**Figure 2:**
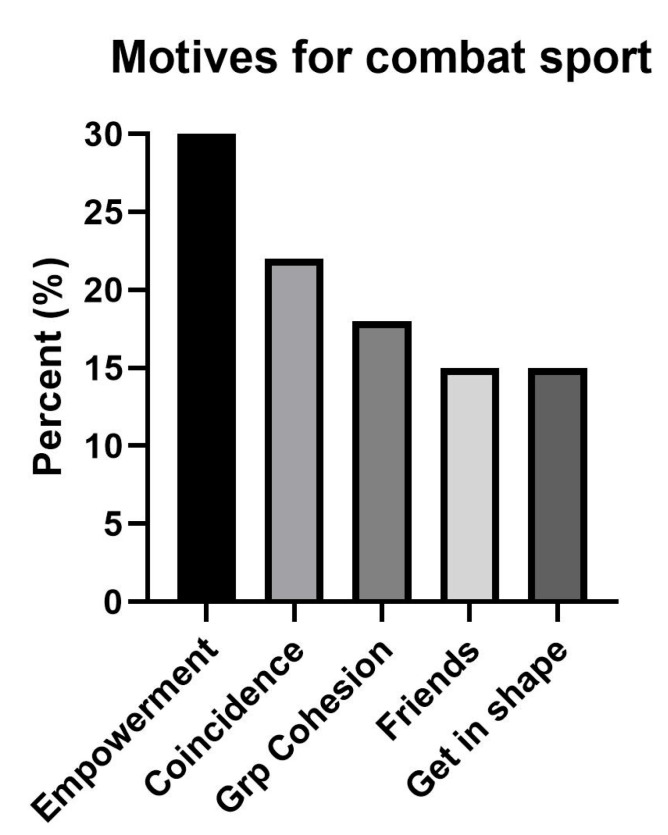
The motives for participating in combat sports. Results are presented as percent of participants who responded to questionnaire (*n* = 27). NOTE: Grp cohesion, group cohesion.

Demographic information on participants is presented in Table 1. The motives for engaging in combat sport are presented in Figure 2. We refer to Figure 1 for an overview of number of participants participating in each of the measurements.

In total, 26 participants (96%) reported in our questionnaire that the COVID-19 pandemic changed their training routines, among which, most reported a reduction in training volume and change in type of exercise (46%), while 19% reported only a reduction in training volume, and 11.5% reported only a change in type of exercise. Additionally, 8% reported to increase their training volume.

The corresponding results for effect on diet revealed that 48% reported dietary change. Among these, eight participants (62%) reported to have increased their energy intake, three participants (23%) reported to have reduced their energy intake, and two (15%) reported to eat healthier.

### 3.1. Mental Health

The mental health characteristics are presented in Table 2. Among the nine (33%) participants who reported a history with/current ED, three reported to have been treated and recovered, and two (reporting no contact with health care or therapy) were still suffering from EDs. Unspecified ED was most frequently reported (*n* = 3, 33%); two (22%) reported a history of anorexia nervosa or bulimia nervosa, respectively; and two (22%) did not know of any specific diagnosis.

In total, seven (26%) scored above the clinical cut-off score on the EDE-q global score, of which five did not report any previous or current ED. Additionally, one participant (4%) reported binge eating, and 11 (41%) reported purging. Driven exercise as the purging method was reported by all of those with a clinical EDE-q global score, with frequency ranging from 4–28 episodes the current month.

Reporting a present ED (β = 0.34, SE = 0.55, 95% CI (0.04, 3.17), *p* = 0.008), higher total exercise volume (β = −0.28, SE = 0.06, 95% CI (−0.30, −0.04), *p* = 0.046), lower BAS-2 score (β = −0.49, SE = 0.27, 95% CI (−0.19, −0.71), *p* = 0.002), and BMI (β = 0.08, SE = 0.61, 95% CI (−0.14, 0.22, −0.71), *p* = 0.555) explained 63.9% of the variance in ED symptoms

### 3.2. Physical Health

Information on body weight and composition is presented in Table 3. The numbers below the recommended Z-score for high impact athletes were two (10.5%) in total BMD, four (21%) in proximal femur BMD, and four (21%) in lumbar BMD. The regression analyses revealed that total exercise volume (β = 0.14, SE = 0.13, 95% CI (−0.31, 0.47), *p* = 0.537), past or present ED (β = −0.46, SE = 0.50, 95% CI (−0.2.60, −0.41), *p* = 0.047), and BMI (β = 0.34, SE = 0.13, 95% CI (−0.18, 0.571), *p* = 0.146) explained 32.2% of the variance in spine BMD Z-scores. No significant regression model was found for total or spine BMD Z-scores.

In total, 18 (67%) reported to have experienced injury, with strains and ruptures most prevalent and a non-attendance to training of more than five weeks being the most typical consequence (*n* = 7, 39%).

The mean (Std) score in LEAF-q was 8.7 (3.8), and in total, 15 (56%) participants had a LEAF-q total score above the cut-off score. While the mean (Std) score in LEAF-q GD subscale was 2.2 (1.4), a total of 17 (63%) scored above the GD cut-off. Among the participants, a number of 10 (37%) did not use any hormonal contraceptives, and their mean (Std) score for LEAF-q subscale MI was 3.1 (2.1), of which 3 (30%) scored above the MI cut-off.

### 3.3. Nutritional Intake, Dietary Information

The nutritional intake of female combat sport athletes is presented in Table 4. In total, 11 (48%) and 20 (87%) had low protein and carbohydrate intake, respectively (i.e., below recommendations). Overall, 21 (88%), 13 (54 %), and 22 (92%) consumed less vitamin D, calcium, and iron, respectively, than recommended.

Among the 27 participants who responded to the questionnaire, 14 (52%) reported to not have received any information on dietary needs as a combat-sport athlete, with seven (26%) being neutral (finding information on their own initiatives) and five (19%) being very positive about the information in their sport (e.g., reporting receiving information and advice and having a club where seminars with nutritional focus are arranged).

### 3.4. Weight Regulation

**Figure 3:**
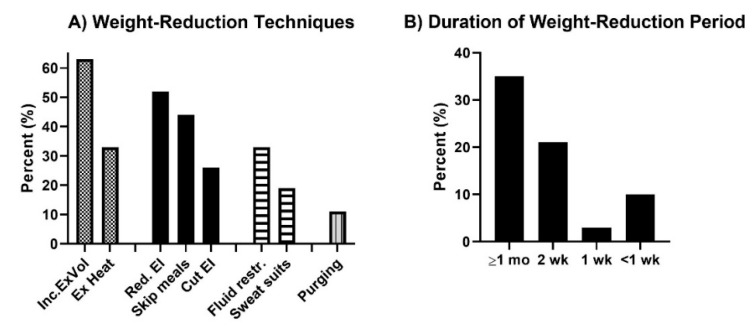
(**A**) Percent of participants who reported the specific techniques for weight reduction and (**B**) the distribution of typical duration of period for weight reduction before competition. NOTE: Inc ExVol, increased exercise volume; Ex Heat, exercising in increased temperatures; Red EI, gradually reduce energy intake; Skipping meals, reducing number of/skipping meals; Cut EI, dramatic cut in energy intake; fluid restr., restricting fluid intake; sweat suits, exercising in sweat suits; purging, using laxatives or self-induced vomiting; mo, months; wk, weeks.

In total, six (22%) reported not to practice any weight regulation. The median (IR) weight loss before competition was 3.0 (5.0) kg. The reported weight-reduction techniques are presented in Figure 3. Most (*n* = 15, 56%) reported to regulate body weight on their own or without receiving any guidance from others, while four (15%) were guided by their coach and two (7%) by a dietitian.

### 3.5. Sexual Harassment

In total, 19 (70%) reported to have experienced SH during THEIR lifetime, 11 (41%) reported to have experienced unwanted SH in a sport context specifically, and 14 (52%) reported such experience outside the sports arena (during leisure time). Among these, seven (26%) had experienced SH in both arenas. Furthermore, five (19%) reported to have experienced SH within the sports arena during the past 12 months on more than one occasion. One of these had experienced SH by a coach, one had been assaulted by a team mate, and three reported “other” to be the perpetrator(s) (i.e., not being a team mate, the coach, or anyone from the medical team).

## 4. Discussion

We aimed to increase our understanding of motives among females to engage in combat sport; explore their experience with weight regulation within the sport and any presence of LEA, DE, or Eds; and to explore any frequency of SH.

Most females reported to engage in combat sport because they wanted to feel empowerment (i.e., be stronger, learn self-defense, become tougher), but many also reported friends or coincidence (e.g., noticing a poster from a club) as reasons. We identified a large frequency of health issues. Between 21–67% of combat sport athletes had symptoms of- and self-reported EDs, suffered from injuries, had low BMD, and/or had symptoms of gastrointestinal dysfunction. We further identified insufficient intake of energy and nutrients and that less than half of the participants received any dietary information or guidance from their clubs. While most combat-sport athletes complied with favorable weight-loss strategies, more than 20% used potentially harmful methods and short weight-reduction periods with rapid weight loss. Finally, we identified that as much as 41% of the athletes had experienced sexual harassment within the combat-sport context.

Engaging in combat sport to be empowered (e.g., to feel safer, to feel confident, to be able to defend one self, to become stronger) involves women who actively challenge the socially constructed gender norms [1,2,3], and who want to feel independent. Additionally, the fact that many argue they practice combat sport because friends motivated them to or because they experience an inclusive milieu points towards a sport that includes women despite such typical gender norms. Nevertheless, while being a friendly milieu, our findings reveal that combat sports need to work on health literacy and educate their leaders, coaches, and the athletes themselves about nutrition, health, and performance in order to avoid negative health effects from combat-sport participation. Few received any information on dietary needs or guidance on weight regulation. The insufficient energy and nutrient intake identified in this study indicate that many athletes are not able to perform at their best or recover properly from training. Fortunately, the most frequent method for weight reduction was a gradual reduction in energy intake with a duration of at least one month. However, many also used dehydration techniques (exercising in heat, fluid restriction, and/or sweat suits) and had rapid and short weight-regulating periods. Our results echo previous findings [10] and reveal an unhealthy culture in combat sports, which needs to be addressed, as many applied weight-regulating behaviors are potentially harmful to health (i.e., dehydration, fluid restriction, and purging methods such as laxatives and diuretics) [31,32]. An interview with combat-sport athletes revealed a strong cultural identity linked to such practices, emphasized the mental importance of achieving the feeling of being a “real athlete” and experiencing increased focus before competition [33]. Concurrently, findings from a study on coaches’ attitudes and recommendations for weight regulation are concerning. Here, weight reduction before competition, including behavior such as different purging methods, was recommended by one of five coaches and advised to athletes from 12 years of age [34].

Despite the knowledge that athletes in weight-sensitive sports are more prone to EDs, females in combat sport are less studied. In our study, almost half of the athletes had either a history or current symptoms of EDs, which is worrying and considerably higher than previous findings [35]. Being cross-sectional in nature, this study is not in position to suggest any causational nature. Still, it may be reasonable to speculate if this is a sport attracting females aiming to improve low self-confidence and who believe that tough exercise with large energy expenditure and normalization of frequent weight regulation will help them in taking control over body weight and figure, which are the core features of EDs. Results from the regression analysis revealed that reporting a current ED, lower total exercise volume, and less body acceptance acted as significant independent explanatory factors for more ED symptoms measured by the EDE-q. Due to low statistical powers and possibly overestimation of the explained variance due to small sample size, these results should be interpreted with caution. Nevertheless, our results show that combat-sport athletes with larger exercise volume have fewer symptoms of EDs, which may be explained by the motive to engage in combat sports. One possible explanation could be that athletes devoted to the sport, who put much effort to increase their performance by exercising more, are less likely to suffer from DE or EDs. On the contrary, those who are attracted to the sport not by sport competitive motives and as such do not put similar high efforts into performance development (i.e., exercising often) are more likely to have symptoms of EDs.

The BAS-2 measures body acceptance, and the mean score is comparable high to recent findings in national comparable age- and sex-matched samples [17,36,37]. As much as body dissatisfaction increases the risk for an ED [38], high body acceptance may protect against DE and EDs [39]. In the current group of athletes, high individual mean scores contributed to the positive finding of high body acceptance. Concurrently, about one-third had poor body acceptance results and a noticeably higher intensity in symptoms of EDs.

A previous study found more favorable eating habits, mental health attitudes, and preventive health practices among athletes in boxing, karate, and MMA compared to Thai boxing [40]. However, they did not take into consideration the EA. In our sample of athletes, the average reported volume of exercise was 10.5 h per week, and the mean energy intake was ~30 kcal per kg body mass. This indicates that many of the current athletes may have experienced a state of LEA (i.e., where an energy availability after expenditure from exercise is < 30 kcal/kg lean body mass). Worth noting is that the data collection in this study originates from a period of societal restrictions (COVID-19 pandemic), and as the athletes concurrently reported that the COVID pandemic meant a decrease in training volume and an increase in energy intake, this points towards a potentially even worse ratio between exercise volume and energy intake in more normal periods. The measure we made of habitual physical activity with the activity devices underscores that the activity level of these athletes are higher than normative values from the general population despite the COVID-19 pandemic period (CPM in current study was 457 and normative in a non-COVID-19 pandemic period is 349 CPM) [41]. Unfortunately, we did not directly measure energy expenditure and are left without any proper evaluation of the actual EA. However, adding to our worries on EA is the finding that almost half of the athletes practiced skipping meals during weight-loss periods. Previous studies have found that adjusting EA proportionally within a day is necessary to avoid negative health effects in both males and females [42,43]. Supporting the suggestion of high frequency of LEA in this sample is the symptom frequency of LEA measured by the LEAF-q (i.e., 56% with symptoms). Unfortunately, we were unable to perform linear regression analyses with LEAF-q scores as dependent or independent variables due to the low α values of LEAF-q scores in our sample.

With regards to body composition, the mean BMI and body fat percentages in our sample were comparable to previously reported levels for female combat-sport athletes at different competitive levels [44,45]. However, results on BMD in female combat-sport athletes are less frequently reported in the literature. High- and odd-impact sports have a potential positive advantage of strengthening bone mass [25,28,46,47,48]. Still, our results indicate that as much as 32% of the athletes had bone mass below the expected level in the lumbar and/or proximal femur region, respectively. Reasons for low BMD are multiple, including genetics, but based on our findings, there is a need for further exploration. In our study, lower spine BMD was significantly explained by having a history of Eds, which is in line with previous research finding increased risk for low BMD if having previous or active ED [49]. Nevertheless, as we identified high frequency of LEA and low intake of vitamin D, candidate explanations occur, as these are important explanations to why athletes may have low BMD [28,50]. The low intake of vitamin D raises additional concerns, as this athlete sample spends much time in indoor training facilities and lives at a geographical location in the northern hemisphere (i.e., deprived from optimal sun exposure).

Our speculation on whether combat sport features a milieu in which SH may easily occur was somewhat supported. A very high number of female combat-sport athletes reported a lifetime experience of SH, with about half of these reporting sport to be the context for their experiences. It is not clear if the young age of these athletes could be considered a risk factor for SH experiences, as previous findings in athletes have been inconsistent [51,52]. The conceptualization of SH in previous studies varies considerably, which complicates comparison. However, our finding is somewhat concurring with previous findings of SH within the sport context [53,54] but also in line with previous studies suggesting that SH is more common during leisure time than in the sport context [54,55]. We are not in a position to explain the higher frequency of SH during leisure time, but one possible explanation supported by the current reported main motives for engaging with this sport (i.e., empowering oneself) may be the previously reported higher vulnerability for SH among persons with low assertiveness [8]. Another reasonable explanation for experience of higher frequency of SH among athletes during leisure time may be that persons outside the sport perceive the female combat-sport athletes as open to sexual invitations due to the typical nature of their sport (i.e., physical and intimate interactions). Even if SH occurred more frequently in leisure time, the high number of SH in the sport context warrants increased attention for implementation of preventive strategies and measures to safeguard athletes experiencing SH.

### Strengths and Limitations

Several strengths of this study are present. We used validated questionnaires to assess LEA and mental health outcomes. We also used categories of weight-regulation strategies similar to what has previously been used [31], allowing for comparison between studies. The study also used objectively measured physical activity as well as the gold standard for estimating BMD (DXA). While not having any definite numbers on female combat-sport athletes within the capital of Norway, we unfortunately suspect the response rate was low. This may imply biased results, as responders may have been specifically motivated by the aim of this study. Nevertheless, most findings in the current study have support from the published literature. The low power following a low sample size was a major limitation with respect to the linear regression analyses, as it limited the number of possible independent variables. The small sample size together with a relatively high number of independent variables may also have resulted in an overestimation of the explained variance and underestimated the SE increasing the risk of type I error [56]. Further, low internal consistency of the LEAF-q made it not possible to examine if symptoms of low energy availability explained BMD and ED symptoms and to examine possible explanatory factors for low energy availability. The low response rate may have been caused by the COVID-19 pandemic, as all training facilities were closed (making it difficult to recruit participants). Because the COVID-19 pandemic was increasing in intensity during autumn 2020, with different national and regional restrictions and social distancing, the different measures for outcomes were differently shifted with regards to sampling time. A recent study reported on weight gain in combat-sport athletes during lockdown and social distancing, and as such, our findings in body weight and composition may have been biased by the extraordinary situation [57]. Furthermore, the dietary registration is based on self-report and may be flawed by both underreporting and changes in the normal diet to ease the registration. In order to reduce underreporting biases, we instructed the participants to submit pictures of their meals in addition to their own registration. Finally, the results on the experience of SH should be interpreted with caution, as the lockdown is likely to have resulted in less time spent in their usual sport setting.

## 5. Conclusions

While many female combat-sport athletes are motivated to increase their empowerment, many started their participation in combat sports because of their friends or by coincidence. Our results underline the urgent need for interdisciplinary actions to safeguard female combat-sport athletes’ mental and physical health and to work towards a cultural change within the community of combat sport with regards to interpersonal relationships, SH, and attitudes towards weight regulation and nutritional knowledge.

## Figures and Tables

**Table 1:** Demographic information. Values are mean (StD) if not otherwise stated.

	Female Combat-Sport Athletes
Age, years ^#^	23.5 (6.3)
BMI, kg × h^−2^	22.7 (1.7)
Combat sport exercise volume, hours per week	6.3 (1.8)
Age of combat-sport specialization, years ^#^	16.0 (11.0)
Exercise volume, other sports, hours per week	4.3 (1.8)
CPM *	457.3 (204.2)
Educational level ≥ BSc, *n* (%)	17 (59)

NOTE: ^#^ median (IR); BMI, body mass index; CPM, counts per minute, a measure of total physical activity; * measured during COVID-19 pandemic lockdown; BSc, bachelor in science degree.

**Table 2:** Mental health characteristics.

	Female Combat-Sport Athletes
Eating disorders, *n* (%)	9 (33)
EDE-q global score ^#^	1.1 (2.1)
EDE-q weight concern ^#^	1.6 (3.0)
EDE-q shape concern ^#^	1.9 (2.8)
EDE-q eating concern ^#^	0.2 (1.0)
EDE-q eating restriction ^#^	1.2 (1.6)
BAS-2 total score	3.8 (0.6)

NOTE: EDE-q, eating disorder examination questionnaire, BAS-2, body appreciation scale−2; ^#^ median (IR).

**Table 3:** Body weight and composition. Values are mean (StD) if not otherwise stated.

	Female Combat-Sport Athletes
Body weight, kg	62.1 (6.4)
Maximal adult BW difference, kg	10.5 (6.0)
Competitive BW, kg	61.5 (7.8)
Body fat percentage (%)	25.4 (5.2)
BMD total body, gram × cm^−2^	1.23 (0.1)
BMD Z-score total body	1.5 (1.1)
BMD proximal femur, gram × cm^−2^	1.1 (0.1)
BMD Z-score proximal femur	0.8 (1.0)
BMD lumbar spine, gram × cm^−2^	1.3 (0.2)
BMD Z-score lumbar spine	0.9 (1.2)

Note: BW, body weight; BMD, bone mineral density; Body composition results are based on 19 of 29 athletes who met for physical screening.

**Table 4:** Nutritional intake by female combat-sport athletes and the national (na) or international sport-specific (sr) recommendations. Values are median (IR).

	Female Combat-Sport Athletes	Recommendations
Energy intake, kcal	1770.9 (467.9)	
Energy intake, kcal × kg BW^−2^	29.8 (10.7)	≥40 kcal × kg LBM^−2^ (sr)
Protein intake	76.0 (42.0)	
Protein intake, kcal × kg BW^−2^	1.4 (0.6)	1.2–2.0 g × kg BW^−2^ (sr)
Carbohydrate intake	201.7 (54.2)	
Carbohydrate intake, kcal × kg BW^−2^	3.4 (1.3)	5–7 g × kg BW^−2^ (sr)
Dietary fiber intake, gram per day	24.6 (10.7)	25 35 g × day^−2^ (na)
Fat intake, percent of energy intake (%)	36.9 (5.7)	25–40 E% (na)
Vitamin D, µg per day	4.4 (5.9)	10 µg (na)
Calcium, gram per day	776.5 (329.8)	800 mg (na)
Iron, mg per day	9.5 (5.7)	15 mg (na)

NOTE: BW, body weight; LBM, lean body mass; na; national recommendations are from the Norwegian Directorate of Health [29]; sr; international sport-specific nutrition recommendations are from the American College of Sports Medicine [30].

## Data Availability

Data can be given on reasonable request.
